# Clinical implications of germline mutations in breast cancer genes: *RECQL*

**DOI:** 10.1007/s10549-018-05096-6

**Published:** 2019-01-04

**Authors:** A. Ramsay Bowden, Marc Tischkowitz

**Affiliations:** 1 East Anglian Medical Genetics Service, Cambridge University Hospitals NHS Trust, Cambridge, UK; 2 Academic Department of Medical Genetics, NIHR Cambridge Biomedical Research Centre, University of Cambridge, Box 238, Level 6 Addenbrooke’s Treatment Centre, Cambridge Biomedical Campus, Cambridge, CB2 0QQ UK

**Keywords:** Breast cancer, Gene panel testing, Topoisomerase inhibition, RECQ, Cancer predispostion

## Abstract

**Background:**

The identification of new hereditary breast cancer genes is an area of highly active research. In 2015, two independent studies provided initial evidence for a novel breast cancer susceptibility gene, *RECQL*, a DNA helicase which plays an important role in the DNA damage response. Several subsequent studies in independent patient cohorts have provided further data on *RECQL* variant frequency in additional populations, some of which have brought in to question the increased breast cancer risk associated with *RECQL* mutations.

**Results:**

The initial reports present findings from whole exome sequencing of high-risk familial breast cancer cases in the French-Canadian, Polish and Han Chinese populations and estimate the carrier frequency of pathogenic *RECQL* mutations in high-risk breast cancer patients who have previously tested negative for *BRCA1* and *BRCA2* mutations to be approximately 1–2%. Proposed founder mutations were identified in French-Canadian and Polish populations. Functional studies support loss of function of the helicase activity of *RECQL* for some of the reported pathogenic mutations. An additional study in a cohort of Southern Chinese high-risk breast cancer patients estimated the frequency of pathogenic *RECQL* mutations to be 0.54%. A possible Chinese founder mutation was identified, but only a small number of controls were sequenced. Subsequent case–control studies screening for the Polish founder mutation in patients from Germany and Belarus did not find any evidence for increased breast cancer risk for this variant. An Australian case–control study also failed to identify an increased risk of breast cancer associated with *RECQL* loss of function variants.

**Conclusions:**

RECQL plays an important role in DNA repair, and is a plausible candidate breast cancer susceptibility gene. Initial studies showed evidence of an association between variants in this gene and an increased breast cancer risk in three separate populations, and identified founder mutations with significantly increased odds ratios. However, several subsequent studies have failed to support the association. With the limited and conflicting evidence available, there remains debate as to whether there is an increased breast cancer risk in individuals carrying *RECQL* loss of function variants. Further studies are required to better quantify the risks associated with *RECQL* variants and the current evidence base is not sufficient to justify routine inclusion of *RECQL* on breast cancer gene panels in clinical use. Management of patients in whom *RECQL* variants have been identified should be based on clinician assessment, in the context of the family history. Further studies are required to better quantify the risks to *RECQL* mutation carriers and may also guide management and potential therapeutic targeting for patients.

## Introduction

*RECQL* (also known as *RECQL1* and *RECQ1*) is located on chromosome 12p12 and encodes a DNA helicase. It belongs to a class of DExH-containing helicases which have important functions in DNA repair, replication, recombination and transcription. In particular, the importance of *RECQL* has been identified in DNA double-strand break repair. The observation that genes encoding other components of this network are known to act as breast cancer susceptibility genes pointed towards the significance of *RECQL* as a potential breast cancer susceptibility gene.

In 2015, two independent research groups who used different screening strategies to search for new cancer predisposition genes, published evidence suggesting that *RECQL* is a novel breast cancer susceptibility gene. Cybulski et al. [1] used whole exome data from patients with high-risk hereditary breast cancer in two distinct populations, Polish and French-Canadian, both known to harbour founder mutations in other hereditary breast cancer genes. Sun et al. [2] used whole exome sequencing in a much smaller cohort of early onset breast cancer patients derived from the Han Chinese population. Both groups went on to perform validation in a broader patient group.

Following these initial studies, further papers were published investigating the frequency of *RECQL* variants in additional breast cancer patient populations. Kwong et al. [3] screened a cohort of breast cancer patients of Southern Chinese origin for *RECQL* variants by targeted sequencing of all coding exons. Nguyen-Dumont et al. [4] used a similar strategy to screen breast and ovarian cancer patients from Poland and Ukraine. Li et al. [5] performed similar screening in a cohort of Australian patients with a family history of breast cancer and a personal history of breast (> 95%) or ovarian cancer. Li et al. additionally sequenced a large control population. Other screening strategies included searching for rare *RECQL* missense variants, predicted to be deleterious, as performed by Tervasmäki et al. [6] in a cohort of Finnish breast cancer patients and controls. Bogdanova et al. [7] screened two separate breast cancer case–control series, one from Belarus, the other from Germany, only for the putative Polish founder mutation (c.1667_1667 + 3delAGTA) identified by Cybulski et al. Table 1 summarises the methodology and variant frequencies across the key studies.

**Table 1:** Comparison of major studies investigating *RECQL* variant in relation to breast cancer susceptibility

Study	Cybulski et al. (2015) [1]	Sun et al. (2015) [2]	Kwong et al. (2016) [3]	Bogdanova et al. (2017) [7]	Nguyen-Dumont et al. (2018) [4]	Tervasmäki et al. (2018) [6]	Li et al. (2018) [5]
**Population studied**	French-Canadian and Polish	Northern Chinese	Southern Chinese	Byelorussian and German	Polish and Ukrainian	Finnish	Australian
**Cases**	Discovery phase—144 Polish and 51 French–Canadian high risk breast cancer patients First validation phase—475 Polish and 475 French-Canadian familial breast cancer patients Second validation phase—13136 Polish unselected breast cancer patients and 538 French-Canadian high risk breast cancer patients	Discovery phase—9 high risk breast cancer patients Validation phase—439 familial breast cancer patients	1110 familial and high risk breast cancer patients	Belarus cohort—1502 largely unselected breast cancer patients German cohort—1124 unselected breast cancer patients	338 breast cancer patients and 89 ovarian cancer patients	Discovery phase—189 familial breast cancer patients Validation phase—1744 unselected breast cancer patients and 202 familial breast cancer patients	4536 women with breast (> 95%) or ovarian cancer and a family history of breast cancer
**Controls**	Discovery phase—4300 exome database controls Second validation phase —4702 adult cancer-free Polish females and 7136 French-Canadian newborns	1588 healthy female controls	88 healthy controls	Belarus cohort—1202 healthy controls German cohort—932 healthy female controls	None tested	Validation phase—1408 cancer-free controls	4576 cancer-free female controls > 40 years of age
**Methodology**	Discovery phase - whole exome sequencing First validation phase—targeted sequencing of *RECQL* coding exons Second validation phase—genotyping for putative Polish founder mutation (c.1667_1667 + 3delAGTA) and putative French-Canadian founder mutation (c.643C > T) in respective populations	Discovery phase - whole exome sequencing Validation phase—targeted sequencing of *RECQL* coding regions	Targeted sequencing of *RECQL* coding regions and exon–intron boundaries	Direct mutation analysis of the putative Polish founder mutation (c.1667_1667 + 3delAGTA) only	Targeted sequencing of *RECQL* coding exons and proximal exon–intron boundaries	Discovery phase – targeted sequencing of 796 DNA damage response genes Validation phase – sequencing of (c.T468G) variant only	Targeted sequencing of *RECQL* coding regions and exon–intron boundaries
**Reported prevalence of RECQL variants**	Discovery phase—truncating variants identified in 5/195 (2.6%) cases and 8/4300 (0.2%) controls Validation phase—Polish founder mutation in 30/13136 (0.23%) cases and 2/4702 (0.04%) controls French-Canadian founder mutation in 7/1013 (0.69% cases) and 1/7136 (0.014%) controls	‘Plausibly pathogenic’ mutations in 9/448 (2.0%) cases and 1/1588 (0.0006%) controls	‘Predicted pathogenic’ variants in 6/1110 (0.54%) cases and 0/88 controls	Belarus cohort—Polish founder mutation in 5/1475 (0.34%) cases and 4/1202 (0.33%) controls German cohort—Polish founder mutation in 4/1121 (0.36%) cases and 2/930 (0.22%) controls	No loss of function mutations. Predicted pathogenic missense mutation in 1/427 (0.002%) cases	Discovery phase—c.T468G variant identified in 1/189 (0.005%) familial breast cancer patients Validation phase -c.T468G variant identified in 6/1946 (0.30%) breast cancer patients and 0/1408 controls	Loss of function variants detected in 13/4536 (0.29%) cases and 25/4576 (0.55%) controls. Rare missense variants in 54/4536 (1.19% cases) and 37/4576 (0.81%) controls

## Results

### Prevalence of mutations

In their discovery phase, Cybulski et al. used whole exome data from 195 patients who had previously tested negative for founder mutations in other key breast cancer susceptibility genes (*BRCA1, BRCA2, CHEK2, NBN* and *PALB2*). They identified five patients carrying truncating *RECQL* mutations (2.6%) compared to 8/4300 (0.2%) in their control cohort (*p* = 2.0 × 10^−4^). They also identified four patients carrying missense variants with a mean allele frequency (MAF) < 1%, although no functional data to determine the pathogenicity of these variants were reported. In the validation phase, two further truncating mutations (both of which were identified as founder mutations—see below), and twelve missense variants were identified.

Using their initial study population of nine high-risk breast cancer patients, who had previously tested negative for *BRCA1* and *BRCA2* mutations, Sun et al. identified *RECQL* mutations in two patients, prompting them to further investigate the gene. Subsequent screening of *RECQL* in 439 familial breast cancer patients (who had tested negative for *BRCA1* and *BRCA2* mutations) found nine patients with putative pathogenic mutations (three nonsense, one splice site and five missense mutations predicted to be pathogenic) giving an overall frequency of 2.0% (0.9% if missense variants are excluded). Only one plausibly pathogenic missense mutation was found in 1588 controls (*p* = 9.14 × 10^−6^).

Kwong et al. screened 1110 breast cancer patients recruited through the Hong Kong Hereditary and High-Risk Breast Cancer Program who had previously tested negative for *BRCA1, BRCA2, TP53* and *PTEN* mutations. They identified four predicted pathogenic mutations in six patients (one frame-shift deletion, two splice site variants and one nonsense variant). Additionally, 14 missense variants were detected; two missense mutations in the RQC domain, identified in three patients, were predicted to be pathogenic by SIFT and PolyPhen, but no functional investigations were reported. Kwong et al. calculated the overall pathogenic mutation frequency in their cohort as 0.54% (6/1110), excluding the missense variants. No predicted pathogenic mutations were identified in the 88 controls tested.

Nguyen-Dumont et al. screened 338 breast cancer patients and 89 ovarian cancer patients and did not identify any loss of function *RECQL* variants. One predicted pathogenic missense variant was identified. No control samples were tested. Li et al. screened 4536 women who had previously had a negative result from *BRCA1* and *BRCA2* mutation testing, had a family history of breast cancer and a personal history of breast cancer (> 95%) or ovarian cancer. They also screened 4576 cancer-free female controls. Thirteen loss-of-function variants were detected in cases (0.29%), compared to 25 loss-of-function variants in controls (0.55%). The difference between cases and controls was not significantly different [Odds ratio (OR) 0.52, 95% CI 0.25–1.06, *p* = 0.072]. Rare missense variants with a minor allele frequency $\le$ 0.5% were detected in 54 cases (1.19%) and 37 controls (0.81%), *p* = 0.073, and there remained no statistically significant difference when the variants were filtered for pathogenicity using *in silico* tools. Notably, a single truncating mutation, c.1859C>G (p.Ser620*) was the most common mutation in both cases (6/13) and controls (16/25) in the Australian study. This mutation is seen at a frequency of 0.20% in the Exome Aggregation Consortium (ExAC) European dataset, although data are present for only a subset of ExAC particpants [8]. Cybulski et al. removed this variant from their analysis of population level data, stating that as the variant falls in the last exon on the protein it is ‘not expected to be deleterious to RECQL protein function’.

Taken together, these studies show frequencies of *RECQL* loss of function variants of 0–2.6% in patients with high-risk familial breast cancer who have previously tested negative for mutations in *BRCA1*/*BRCA2* and other hereditary breast cancer genes. The frequency of loss of function variants in the control cohorts of these studies range from 0 to 0.55%.

### Founder mutations

The data analysed by Cybulski et al. was specifically selected from populations known to harbour founder mutations. The identification of founder mutations in both the French-Canadian and Polish populations was an outcome of the validation phase of the study. One recurrent truncating mutation: c.643C > T (p.Arg215) was identified in the French-Canadian population in 7/1013 (0.69%) high-risk breast cancer patients compared to 1/7136 (0.014%) controls (*p* = 3.0 × 10^− 6^). The founder mutation in the Polish population: c.1667_1667 + 3delAGTA (p.K555delinsMYKLIHYSFR), was seen in 30/13136 (0.23%) breast cancer cases and 2/4702 (0.04%) cancer-free controls (*p* = 0.008).

Further data on the Polish founder mutation identified by Cybulski et al. were provided by Bogdanova et al. who undertook c.1667_1667 + 3delAGTA mutation analysis in two breast cancer case–control series from Belarus and Germany. In the Belarus cohort of 1475 breast cancer patients and 1202 healthy controls, the mutation was identified in five cases (0.34%) and four controls (0.33%). In the German cohort of 1121 breast cancer patients and 930 healthy controls, the mutation was identified in four cases (0.36%) and two controls (0.22%). Combined analysis of these two case–control series did not confirm the association of this *RECQL* mutation with a significantly increased risk of breast cancer (Mantel–Haenszel OR 1.23, 95% CI 0.44–3.47; *p* = 0.69). Although the frequency of the mutation in breast cancer cases is similar to that seen in the study from Cybulski et al., the frequency of the mutation in the control populations is much higher (0.2–0.3% vs. 0.04%, *p* = 0.01). By comparison, frequency of this mutation in the ExAC non-Finnish European dataset is 0.06%. This mutation was not identified by Nguyen-Dumont et al. in any of the 304 Polish or 123 Ukrainian patients in their cohort.

The possibility of a Chinese *RECQL* founder mutation was raised by Kwong et al. who identified two patients carrying the c.796C > T (p.Gln266*) variant, which was also detected in one patient in the earlier study by Sun et al. This variant was also detected by Li et al. in one control subject in their Australian cohort, but was not detected in any cancer patients in their study. Notably, this variant is also present in five individuals of East Asian origin in the ExAC dataset, giving frequency of 0.06% in this population. Tervasmäki et al. suggest a possible Finnish *RECQL* founder mutation, identified by their search for rare *RECQL* missense variants. In a cohort of 1946 breast cancer cases, they observed six carriers of the missense variant c.468T > G (p.I156M) (0.3%), which was not identified in any of the 1408 controls tested, giving a borderline significance (*p* = 0.043).

### Penetrance of breast cancer

None of the studies comment on the penetrance of breast cancer associated with *RECQL* mutations, but information can be drawn from the odds ratios (OR) presented [9]. Li et al. do not report a significantly increased risk of breast cancer in their analysis of all loss of function mutations in the Australian case–control cohort. (OR 0.52, 95% CI 0.25–1.06; *p* = 0.072). However, Cybulski et al. report an odds ratio of 5.4 for the Polish founder mutation data, with very wide 95% confidence intervals (1.3–46). Data from Bogdanova et al. taken alone, does not show a significantly increased risk of breast cancer associated with the Polish founder mutation (Mantel–Haenszel OR 1.23, 95% CI 0.44–3.47; *p* = 0.69), but a meta-analysis combining their data with data from Cybulski et al. gives an overall odds ratio of 2.51 (95% CI 1.13–5.57; *p* = 0.02). These data relate only to the c.1667_1667 + 3delAGTA mutation, which is a splicing mutation, predicted to result in insertion of 27 nucleotides disrupting a crucial hairpin sequence. These data suggest the possibility that the Polish founder mutation is associated with an increased breast cancer risk, and it could be that breast cancer risks associated with *RECQL* mutations are highly variant specific. More data are needed to confirm whether the Polish founder mutation is associated with an increased breast cancer risk and determine the risks associated with other mutations.

### Range of cancer sites implicated

Cybulski et al. report that two *RECQL* truncating mutation carriers (not carriers of the putative founder mutation) had a positive family history of ovarian cancer. Tervasmäki et al. report a family history of ovarian cancer in three of the six patients carrying the p.I156M missense variant, and pancreatic cancer in two families. Sun et al. provide further information on a single family in which a *RECQL* splicing mutation 395-2A > G was identified. Twelve individuals in this pedigree were affected with cancer: seven breast, one ovarian, one peritoneal, one cervical, one lung and one malignancy of uncertain origin. In the second Chinese cohort of mutation carriers, Kwong et al. report one patient with a family history of prostate and colon cancer, and two patients with a family history of liver cancer. No clear pattern of additional associated cancer sites is apparent from currently available data on *RECQL* mutation carriers and associated family history.

### Determining the pathogenicity of variants

The plausible role of *RECQL* as a pathogenic gene was theorised by all groups. *RECQL* is one of the five *RECQ* genes, three of which (*BLM, WRN* and *RECQL4*) have been implicated in genetic disorders with increased cancer risk [10–12]. It has also been proposed that variants in *RECQL5* are associated with increased breast cancer risk in the Chinese population [13]. However, prior to the potential association with breast cancer susceptibility, mutations in *RECQL* had not previously been reported in any human genetic disorders.

*RECQL* is involved in double strand break repair and plays an important role in the maintenance of genomic stability. *RECQL* prevents dsDNA breaks by stabilising stalled or regressed replications forks [14]. It is involved in non-homologous end joining [15] and lengthening of telomeres without telomerase [16]. The protein product contains two important domains: the helicase domain and the RecQ carboxy-terminal domain. Both are highly conserved in the RECQ family and are important for helicase activity.

In vitro work on *RECQL* has been carried out in both mouse and human cell lines. Primary embryonic fibroblasts from RECQL-knock down mice showed aneuploidy, spontaneous and frequent chromosomal breakage and translocations [17]. Mouse and human RECQL-depleted cells display high rate of spontaneous sister chromatid exchange and are more sensitive to ionising radiation [18].

Ahmed et al. argue that observed differences in breast cancer risk between studies may be due to the fact that only certain variants are pathogenic [19]. In terms of specific variants, Cybulski et al. provide *in silico* data to predict the pathogenicity of the recurrent Polish mutation that they identified. This in-frame indel displaces residues 558–566 which form a beta hairpin in the secondary structure of the *RECQL* protein. This structural hairpin functions as a DNA strand separator and is likely to be critical to the protein’s function in unwinding the replication fork [20]. Tervasmäki et al. hypothesise the pathogenicity of the p.I156M missense variant based on evolutionary conservation of this region, and localisation to the ATPase domain.

Sun et al. provide a more detailed analysis of some of their variants. Fifteen variants were identified in their final cohort of 448 patients (three nonsense, two potential splice site and ten missense mutations). Three missense variants were also identified in the control population and two were presumed not to be pathogenic due to their similar frequency in cases and controls. In vitro functional studies using GST fusion proteins showed that five out of eight missense variants significantly disrupted helicase activity. Splice site analyses by RT-PCR provided evidence of potential pathogenicity for only one of the two splice site variants. All three nonsense mutations were found to lie within the helicase domain, and their effect of premature protein termination resulting in loss of helicase activity led the authors to class them as pathogenic variants, although the functional data to support this is not presented. Sun et al. conclude that nine of the original 15 variants they identified (three nonsense, one splice site and five missense mutations) are likely to be pathogenic. This number may be an overestimate given that pathogenicity was determined by a single functional assay and other supporting investigations, such as segregation analyses, are not reported.

### Clinical management of women without cancer with a mutation

The average age of breast cancer diagnosis for *RECQL* mutation carriers is reported in both initial publications and follow-up studies. Cybulski et al. report a mean age of diagnosis of 54.5 years in *RECQL* mutation carriers in the Polish population and 48.9 years in the Canadian population. In the German cohort analysed by Bogdanova et al. the mean age of diagnosis for *RECQL* mutation carriers was 51 years, and in the patients from Belarus the mean age of diagnosis was 46 years. Sun et al. report a mean age of onset of breast cancer of 45.1 years in the patients in whom pathogenic *RECQL* mutations were identified (range 31–71 years), and on analysis of pedigrees of affected family members the average age of breast cancer diagnosis was 47.8 years. Kwong et al. report a mean age at diagnosis of 48.9 years in patients carrying a *RECQL* mutation. Carriers of the p.I156M missense variant identified by Tervasmäki et al. were diagnosed at a mean age of 60 years. In none of the analyses was there a statistically significant difference in age at diagnosis between *RECQL* mutation carriers and controls.

Testing for hereditary breast cancer genes is increasingly carried out using cancer gene panels. Deciding which genes and variants should be included on such panels is a subject of much debate [21–24]. When designing gene panels for routine clinical use, careful review of the data for each gene should be undertaken to determine whether the test result will be of clinical utility in the management of that patient and/or their family members. When counselling patients it is important that reliable information on relative or absolute breast cancer risk can be provided, which will guide screening and management advice. For recently identified susceptibility genes, interpretation of results can prove problematic when novel variants are identified, particularly in ethnic groups in which there is minimal data on population variants, and little functional data is available. Against these limitations, the benefits of expanding such gene panels to include newly identified genes, which may provide valuable information on familial breast cancer cases, and would not previously have been identified, must be balanced. Overall, at present, the significant limitations in the available data on *RECQL* means that testing of *RECQL* outside a research setting should not be undertaken.

Clinical guidance for women in whom a putatively pathogenic *RECQL* mutation is identified can only be based on limited data at present, and consideration of family history and other risk factors should form the major part of the clinical assessment, including the level of breast surveillance to offer. The current contradictory evidence means that one cannot use *RECQL* mutation status to guide any aspect of clinical management such as breast surveillance or consideration of risk reducing mastectomy. Although a family history of ovarian cancer is described in a small number of cases, studies to date have not identified any individuals with ovarian cancer who carry a *RECQL* mutation, and there is no evidence base on which to recommend ovarian cancer surveillance or prophylactic oophorectomy.

## Conclusions

The initial evidence independently put forward by Cybulski and Sun proposed *RECQL* as a novel breast cancer susceptibility gene responsible for a small proportion of hereditary breast cancer. The possibility of founder mutations in the Polish, French-Canadian and Chinese populations was postulated. Data from subsequent studies has provided further information on frequencies of *RECQL* variants in additional case – control cohorts. Much of this work does not support the association between increased breast cancer risk and loss of function *RECQL* mutations.

Variations in methodology, for example, the presence of sufficient matched controls and exclusion of certain variants, may account for some differences between studies. Variants detected at a low frequency in an affected patient group may be viewed as founder mutations, but if these are then found at a similar frequency in an unaffected control population, such claims need to be reviewed. The presence of potential loss of function *RECQL* variants at frequencies > 0.01% in several ethnically distinct populations may indicate a number of founder mutations, each with a small but significant increased risk of breast cancer, or it may be evidence of a lack of association between variants in this gene and increased breast cancer risk. It is also possible that only certain *RECQL* variants are pathogenic or that that *RECQL* mutations are only pathogenic in combination with other susceptibility alleles. With the limited and conflicting evidence available, there remains debate as to whether there is an increased breast cancer risk for women carrying *RECQL* loss-of-function variants.

Further studies are required to better quantify the risks associated with *RECQL* variants. Widening research to a broader population base of hereditary breast cancer cohorts will help to improve the understanding of the global significance of this gene. There are currently insufficient data to warrant inclusion of *RECQL* on hereditary breast cancer gene panels used in routine clinical practice. Management of patients in whom *RECQL* variants have been identified should be based on clinician assessment in the context of the family history, not *RECQL* mutation status.

As more data become available the importance of identifying pathogenic *RECQL* mutations in women with a family history of breast cancer will become clearer. The important role of RECQL in the DNA damage response raises the possibility that identification of *RECQL* mutations might have potential therapeutic implications for women with breast cancer. *In vitro* studies have demonstrated that RECQL-deficient cells are particularly sensitive to topoisomerase inhibition [18]. As in the case of tumours deficient in *BRCA1*/*BRCA2*, the deficient DNA repair in *RECQL*-deficient tumours could increase their sensitivity to other drugs blocking compensatory DNA repair mechanisms [25]. Information on genetic variants could help to identify populations of interest for trials of targeted therapeutics.

Given the conflicting data at present, it is inappropriate to recommend clinical guidelines on screening and management. Further case–control studies in large and ethnically diverse populations are needed to better understand whether there is an increased risk of breast or other cancers associated with specific *RECQL* variants.
